# Trace element and isotope deposition across the air–sea interface: progress and research needs

**DOI:** 10.1098/rsta.2016.0190

**Published:** 2016-11-28

**Authors:** A. R. Baker, W. M. Landing, E. Bucciarelli, M. Cheize, S. Fietz, C. T. Hayes, D. Kadko, P. L. Morton, N. Rogan, G. Sarthou, R. U. Shelley, Z. Shi, A. Shiller, M. M. P. van Hulten

**Affiliations:** 1 Centre for Ocean and Atmospheric Science, School of Environmental Sciences, University of East Anglia, Norwich NR4 7TJ, UK; 2 Department of Earth, Ocean and Atmospheric Science, Florida State University, Tallahassee, FL 32306, USA; 3 LEMAR/IUEM, UMR 6539 CNRS-UBO-IRD-IFREMER, Place Nicolas Copernic, Technopôle Brest Iroise, 29280 Plouzané, France; 4 Department of Earth Sciences, Stellenbosch University, 7600 Stellenbosch, South Africa; 5 Department of Marine Science, University of Southern Mississippi, Stennis Space Center, Kiln, MS 39529, USA; 6 Applied Research Center, Florida International University, 10555 West Flagler St., Engineering Center Suite 2100, Miami, FL 33174, USA; 7 GEOMAR, Helmholtz Centre for Ocean Research Kiel, 1–3 Wischhofstrasse, Kiel 24148, Germany; 8 School of Geography Earth and Environmental Sciences, University of Birmingham, Birmingham, UK; 9 Laboratoire des Sciences du Climat et de l'Environnement (LSCE), IPSL, CEA–Orme des Merisiers, 91191 Gif-sur-Yvette, France

**Keywords:** air–sea exchange, atmospheric deposition, trace element solubility, biogeochemical impacts, mineral dust, anthropogenic aerosols

## Abstract

The importance of the atmospheric deposition of biologically essential trace elements, especially iron, is widely recognized, as are the difficulties of accurately quantifying the rates of trace element wet and dry deposition and their fractional solubility. This paper summarizes some of the recent progress in this field, particularly that driven by the GEOTRACES, and other, international research programmes. The utility and limitations of models used to estimate atmospheric deposition flux, for example, from the surface ocean distribution of tracers such as dissolved aluminium, are discussed and a relatively new technique for quantifying atmospheric deposition using the short-lived radionuclide beryllium-7 is highlighted. It is proposed that this field will advance more rapidly by using a multi-tracer approach, and that aerosol deposition models should be ground-truthed against observed aerosol concentration data. It is also important to improve our understanding of the mechanisms and rates that control the fractional solubility of these tracers. Aerosol provenance and chemistry (humidity, acidity and organic ligand characteristics) play important roles in governing tracer solubility. Many of these factors are likely to be influenced by changes in atmospheric composition in the future. Intercalibration exercises for aerosol chemistry and fractional solubility are an essential component of the GEOTRACES programme.

This article is part of the themed issue ‘Biological and climatic impacts of ocean trace element chemistry’.

## 1. Introduction

A great deal of research activity has focused on addition of material to the ocean across the air–sea interface, because the realizations that iron (Fe) plays a key role as a limiting nutrient for primary productivity or biological nitrogen fixation in large areas of the global ocean [1–3] and that the deposition of mineral dust from the atmosphere was a major source of Fe to the remote ocean [4]. That research has led to huge advances in the understanding of the impact of Fe biogeochemistry on the marine carbon cycle [5], the sources and composition of Fe-bearing material to the atmosphere [6,7] and the chemical and physical processing of that material during transportation through the atmosphere [8]. Alongside those advances has come the understanding that a number of other trace elements (TEs) that are deposited across the air–sea interface (e.g. manganese (Mn), cobalt (Co), zinc (Zn), nickel (Ni), cadmium (Cd), copper (Cu) [9]) have micronutrient functions for marine microbial organisms or have potentially toxic effects (e.g. Cu [10]).

In that context, one of the goals of the international GEOTRACES programme is to extend knowledge of the exchange across the air–sea interface, based on the understanding that mineral dust constitutes a vector for a wider range of important trace elements and their isotopes (TEI) than Fe alone and that the sources of TEI in atmospheric deposition to the ocean are not limited to mineral dust [7,11].

This paper aims to highlight recent progress in this field, with a focus on research driven by the international GEOTRACES programme, and identify topics for which further effort is still required. Two long-standing problems—the difficulties in making accurate estimates of the atmospheric flux of material to the ocean and in determining the fraction of the atmospheric flux of bioactive substances that is available to marine biota—continue to challenge our understanding. The extent to which anthropogenic emissions contribute to the atmospheric flux to the oceans and their biogeochemical response to that flux is also of increasing interest.

## 2. Highlights of recent progress

### (a) Estimation of deposition flux

Atmospheric deposition is an important source of biologically essential TEs to the open ocean. Knowledge of these fluxes helps us understand and model ocean productivity, yet these fluxes are extremely difficult to measure. Although autonomous buoys capable of collecting aerosol samples over extended periods have been developed and deployed at remote ocean sites [12], long-term monitoring of the composition of aerosols and rainfall over the vast majority of the remote ocean is effectively impossible due to limitations associated with the lack of suitable island sampling locations and the expense of ship deployment to areas where island sites are not available. In a few regions, where long-term records from island sampling sites exist (e.g. [13]) or where specific ocean areas are subject to relatively intense research ship activity [14–17], flux estimates based on direct atmospheric sampling can be made. By combining observations of aerosol and rainwater chemistry made during 28 research cruises of the GEOTRACES and other research programmes (such as the Atlantic Meridional Transect (AMT) and the Surface Ocean Lower Atmosphere Study (SOLAS)), Powell *et al.* [16] were able to estimate seasonally resolved 10-year average atmospheric fluxes for 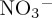 and 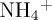 and soluble and total Fe, aluminium (Al) and Mn, for the eastern tropical North Atlantic. However, the uncertainties associated with such deposition estimates are considerable [14–16]. Aerosol dry deposition to the ocean surface cannot be directly measured, necessitating the use of highly uncertain dry deposition velocities to convert measured aerosol concentrations into dry deposition flux. Direct measurements of wet deposition fluxes are hampered by either biases in rainfall patterns (compared to the open ocean) at island sites, or the difficulty in measuring precipitation rates and the collection of sufficient rainfall samples to represent the wet deposition flux effectively from ships.

With trace metals that partially solubilize from mineral dust, such as Al, titanium (Ti), gallium or thorium (Th), one can indirectly estimate dust deposition using the dissolved distribution of these metals in seawater. The use of stable (non-radioactive) tracers to estimate dust fluxes often relies on variations in equation (2.1) (where dissolved Al is used as an example). The concentration of dissolved Al in the surface ocean has been widely used as a dust deposition proxy because Al is abundant in dust (about 8% by mass) and is not biologically essential (e.g. [18–23]):
2.1
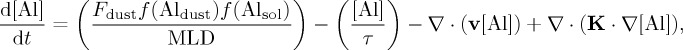

where [Al] = dissolved Al concentration (g m^−3^) in the surface ocean mixed layer, *F*_dust_ = flux of dust (g m^−2^ d^−1^), *f*(Al_dust_) = fraction of Al in dust (typically approx. 0.08 g total Al g^−1^ dust), *f*(Al_sol_) = fraction of soluble Al in dust (variable (see below), but typically assumed to be approximately 0.03 g soluble Al g^−1^ total Al), MLD = mixed layer depth (m), *τ* = residence time of dissolved Al in the MLD (typically of the order of 5 years), ∇ · (**v** [Al]) = effects of advection (in *x*, *y* and *z*) on the concentration of dissolved Al, ∇ · (**K** · ∇[Al]) = effects of turbulent mixing (in *x*, *y* and *z*) on the concentration of dissolved Al.

The dust flux term is assumed to dominate the input of the tracer. The residence time can be separated into components influenced by multiple removal processes such as particle adsorption (scavenging removal) or incorporation into biogenic particles (biological uptake). The removal rates are modelled as first order with respect to dissolved Al. For tracers with short residence times, advection and mixing are often thought to be small and therefore insignificant (the implications of this simplification are discussed below). Assuming steady-state conditions, and neglecting physical mixing and advection, equation (2.1) resolves to
2.2
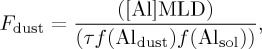


which represents the MADCOW model [24], where the numerator is the inventory of the tracer in the mixed layer. A comparison between this model (applied to dissolved Al and dissolved ^232^Th) and other methods for estimating dust deposition is presented in Anderson *et al.* [23]. This intercomparison also demonstrates very dramatically how different methods for measuring aerosol TEI solubility have a significant influence on the dust flux estimates.

**Figure 1.**
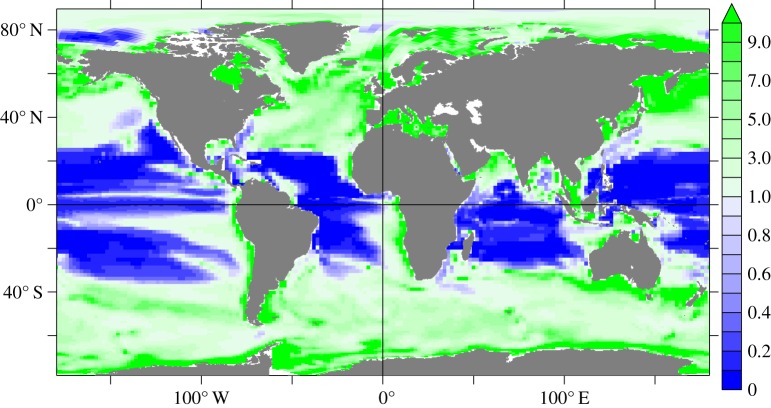
Distribution of the ratio of advection to scavenging timescales (*Y*) for Al in the global ocean (after [26]). This is a measure of the relative importance of scavenging versus advection for Al export. Regions where *Y* is higher than one are scavenging-driven, those where Y is smaller than one are advection-driven. Where advection dominates (blue) the one-dimensional MADCOW model is predicted to be unreliable.

Whenever possible, it is preferable to make use of the full equation (equation (2.1)) and to make measurements that are relevant for the region and time of year. Rates of particle scavenging and uptake into biogenic material can vary from regime to regime and season to season. The depth of tracer penetration can also vary in space and time, as can the sources and chemical nature of the aerosols. The physical transport terms (particularly horizontal advection) may not be insignificant. Van Hulten *et al.* [25,26] showed how important this can be, using a general ocean circulation model to take into account the effects of particle scavenging, biogenic particle uptake and physical transport. These authors compare the timescales (residence times) for dissolved Al in the upper water column with respect to advection and particle scavenging
2.3
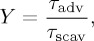

and use this ratio to recommend where one-dimensional models, like MADCOW, might be applied with confidence (when 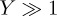, e.g. the North Pacific Ocean and Mediterranean Sea) and where not (when 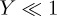, e.g. the equatorial Atlantic Ocean) (figure 1).

Because the fractional solubility of aerosol Al exhibits significant variability (e.g. [27–30]) and because dissolved Al has a somewhat complicated behaviour in the upper ocean (with respect to abiotic and biotic scavenging; e.g. [31]), it has been suggested to use dissolved Ti as an alternative dust input proxy [32].

A tracer that shows promise as a way to estimate atmospheric deposition is the natural radionuclide beryllium-7 (^7^Be: half-life 53.3 days—comparable to the lifetime of particles in the surface ocean [33]; gamma energy 0.4776 MeV). It is produced in the upper atmosphere from cosmic ray spallation, quickly attaches to aerosol particles, and is transported to the lower troposphere by atmospheric circulation processes. Because it is associated with sub-micrometre aerosols, the deposition of aerosol ^7^Be is dominated by rainfall scavenging [34,35]. Given the relatively short half-life of ^7^Be, at steady state the input flux of ^7^Be (atoms m^−2^ min^−1^) is balanced by the ^7^Be inventory, or decay rate, integrated over the upper water column (dpm m^−2^). The important point is that the ability to derive the atmospheric flux of ^7^Be from its ocean inventory provides a key linkage between the atmospheric concentration of chemical species and their deposition to the ocean [34,35].

The flux (*F_*i*_*) of an aerosol element into the ocean can be described as the sum of wet and dry deposition processes, respectively:
2.4
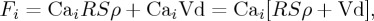

where *F_*i*_* = flux to the oceans (µg m^−2 ^d^−1^), Ca*_*i*_* = aerosol concentration 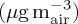, *R* =precipitation rate (m_rain _d^−1^), *S* = washout ratio (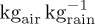; i.e. the concentration in rain 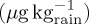 divided by the aerosol concentration 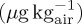), *ρ* = ratio of the densities of water and air 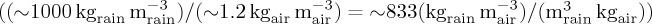, Vd = aerosol dry deposition velocity (m d^−1^).

The bracketed term on the right-hand side of equation (2.4) represents the effective ‘bulk deposition velocity’, combining wet and dry deposition.

The aerosol dry deposition velocity (Vd) to the ocean surface is a function of humidity, wind speed and particle size and has been estimated to vary by a factor of 3 for sub-micrometre aerosol particles [36]. There are also large uncertainties associated with wet deposition estimates [37]. The rain rate over the ocean is very difficult to constrain as direct measurement of patchy and episodic rain events over vast, remote areas is impractical. Remote determinations from, for example, microwave imager and precipitation radar suffer in accuracy (e.g. [38]). However, we can use the known flux of ^7^Be (calculated from the ocean ^7^Be decay inventory) to avoid the pitfalls associated with determination of these parameters.

The ratio of the atmospheric flux of any aerosol component to that of ^7^Be is
2.5
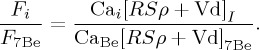


Assuming that the right-hand terms in brackets roughly cancel
2.6
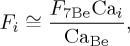

such that the flux of any aerosol component can be estimated by multiplying the ^7^Be flux by the ratio of that component to ^7^Be in aerosols. For many ocean areas [34], this formulation works well because seasonal variation in the aerosol ^7^Be concentrations and the resulting ocean inventory of ^7^Be are small. In regions where there is large seasonal variability in the ^7^Be aerosol concentrations, such as the Arctic Ocean, the expected ^7^Be inventory resulting from the input and decay of aerosol ^7^Be can be described by the following equation [39]:
2.7
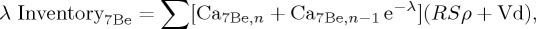

where *λ* is the ^7^Be decay constant (0.013 d^−1^), Inventory_7Be_ is the predicted ^7^Be inventory to the depth of ^7^Be penetration (100–200 m; dpm m^−2^), Ca_7Be,*n*_ = ^7^Be aerosol concentration on day ‘*n*’ 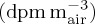, 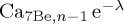 is the ^7^Be aerosol concentration on the previous day corrected for radioactive decay 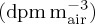.

As in equation (2.4), the right-hand terms in parentheses represent the effective bulk deposition velocity (m d^−1^) that combines precipitation plus dry deposition.

This approach has been used in the central Arctic Ocean, yielding a bulk deposition velocity of approximately 1350 m d^−1^ [39]. For the subtropical North Atlantic, using equation (2.6), a bulk deposition velocity of approximately 2400 m d^−1^ was derived [34]. In both cases, the estimated bulk deposition velocity is higher than the dry deposition velocity that is often used to estimate mineral aerosol dry deposition (1 cm s^−1^ = 864 m d^−1^); this is consistent with the conclusion that ^7^Be deposition is dominated by wet deposition. These bulk deposition velocities from a given region of the ocean can then be used to estimate the flux of any other aerosol component, despite the complication that larger mineral dust aerosols may have higher dry deposition velocities and lower rainfall scavenging ratios. Considering Fe in mineral dust, for example, one might use Vd = 1000 m d^−1^ and a rainfall scavenging ratio (*S*) of 200 [36], while for ^7^Be one might use Vd = 86 m d^−1^ and *S* = 500 [40]. Using these estimates (and equation (2.5)), for a rainfall rate of 4 mm d^−1^, the bulk deposition velocity for aerosol Fe would only be 5% lower than that for ^7^Be [34]. The higher proportions of larger mineral dust particles immediately downwind of sources like the Sahara may impact on the choice of deposition velocities and scavenging ratios for modelling deposition in those regions.

### (b) Trace element solubility

Understanding the fraction of the atmospheric flux of bioactive substances that is available to marine biota is a key part of assessing the biogeochemical impact of that atmospheric flux. Defining the bioavailable fraction is extremely complex, but in many cases the soluble fraction of TE deposition constitutes a major part of the bioavailable fraction [41,42].

In laboratory studies, a positive relationship has been reported between the solubility of aerosol Fe (and other TEs) and aerosol acidity (e.g. [43,44]). These studies were undertaken, in part, to simulate pH changes that occur when aerosol particles cycle through clouds (a process that can also affect other factors that influence solubility, such as aerosol constituent mixing). Despite these results, and there being a general consensus in the community that aerosol chemistry is a key control on aerosol TE solubility, field observations have failed to convincingly reproduce this relationship for the most part. In the Atlantic Ocean, no relationship between acid species, such as non-sea salt sulfate (nss-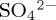) and nitrate 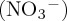, or net potential acidity (i.e. the difference between total acid species concentrations and total alkaline species concentrations) and fractional Fe solubility has been observed [30,45]. By contrast, in the Pacific Ocean, a significant relationship between aerosol acid species, but not oxalate concentration, and soluble aerosol Fe has been observed (e.g. [27]). Similar observations were made of the relationships between aerosol Al solubility and acid species concentrations at Hawaii [46]. This led Buck *et al.* [27] to conclude that aerosol provenance was the dominant control on TE solubility.

There are a number of theories suggested to explain why field data generally fail to capture a relationship between aerosol TE solubility and aerosol acidity. For example, the large buffering capacity of CaCO_3_ means that mineral dust particles do not easily become acidic. The pH of the aqueous solution surrounding dust aerosols is controlled by the ionic balance between acidic species (e.g. sulfate, nitrate, chloride anions) and basic species, including ammonium and components of mineral dust itself, i.e. calcite (CaCO_3_). Before Fe can be effectively mobilized from the particle through proton-promoted dissolution processes [47], the concentration of acidic species must be sufficiently high to overcome the alkalinity of mineral dust (which will vary according to the source and composition of the dust), and decrease the pH of the aqueous solution surrounding the dust particle. Alternatively, part of the problem in linking aerosol acidity and TE solubility may lie in the difficulty of determining the acidity of aerosol particles directly, and therefore proxies are frequently used [48]. Hennigan *et al.* [48] found from their model study that approaches that combined aerosol and gas inputs showed the best agreement with the aerosol pH predicted from the phase partitioning of ammonia, and that ionic balance or molar ratio approaches failed to accurately predict aerosol pH. The highly complex nature of atmospheric aerosol suspensions, in which aerosol components may be fully externally mixed (present in the same volume of air but in different particles), fully internally mixed (present within the same particles within that air volume) or at some point on a continuum between these two extremes, also makes a complete understanding of field observations of TE solubility very challenging. At present, it is not possible to acquire measurements of TE solubility on individual aerosol particles and hence it is not possible to distinguish between observations for which acid species are externally mixed with TE-containing particles and those for which internal mixing might lead to increased solubility.

In the future, in contrast to the ocean, the atmosphere is predicted to become more basic [49]. Emissions of SO_2_ and NO_*x*_ are expected to continue to decline as a result of stricter and/or more commonplace regulation and technological advances, whereas global ammonia emissions (the majority, approx. 80%, of which come from the agriculture sector) are difficult to control and are relatively unchecked [50]. Gaseous ammonia is the most abundant alkaline gas in the atmosphere, and global emissions have increased over the last few decades. A more basic atmosphere might be expected to reduce aerosol TE solubility.

Recent work has highlighted the impact of organic matter on TE speciation and solubility in aerosols and rainfall. Aerosol particles and rainwater are known to contain Fe-binding organic ligands such as formate, acetate and oxalate [51]. These ligands facilitate the dissolution of Fe in aerosol and stabilize soluble Fe [52–57]. Kieber *et al.* [58] estimated that 69–100% of Fe(III) in rainwater was organically complexed. The concentrations of the Fe organic ligands and their conditional stability constants have been directly measured in rainwater only very recently with a new sensitive method using competitive ligand exchange–adsorptive cathodic stripping voltammetry [59]. Ligand concentrations in the first measured samples of coastal rainwater were as high as 336 ± 19 nM, with log 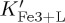 around 21.1–22.8 at pH = 5.45 [59]. These 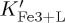 values correspond to the strong ligand class in seawater [60] and imply that 80–100% of Fe in rainwater is organically complexed [59], confirming the estimation of Kieber *et al.* [58]. The presence in rainwater of ligands capable of complexing other TEs, e.g. Cu [61], has also been demonstrated. However, the exact nature and origin of these atmospheric ligands are still largely unknown.

**Figure 2.**
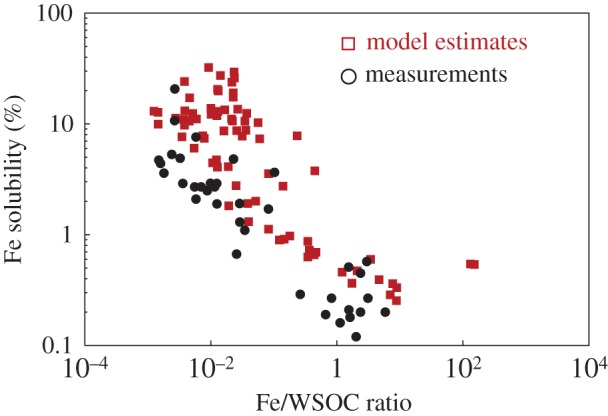
Fe solubility as a function of Fe/WSOC molar ratio for model estimates (red squares) and measurements (black circles, from [62,63]) of aerosol samples collected over the Atlantic Ocean. Reproduced from [53].

The molar ratios of Fe/water soluble organic carbon (WSOC) in aerosols collected during two GEOTRACES cruises were found to be anti-correlated with Fe solubility (figure 2), suggesting a possible role of organic ligands in enhancing Fe solubility [62,63]. Using a global chemical transport model that considered the oxalate-promoted Fe dissolution in aerosols, Ito & Shi [53] successfully reproduced the inverse relationship of Fe solubility and Fe/WSOC ratio over the cruise tracks (figure 2). The process-based modelling by Ito & Shi [53] suggested that proton- and oxalate-promoted Fe dissolution in the aerosol aqueous phase and mixing with combustion aerosols are the main mechanisms to cause the high Fe solubility at low Fe loading in the North Atlantic. This is consistent with observations (e.g. [64,65]) and previous modelling [66].

Primary biological aerosol particles, also called bioaerosols, include fungi, pollen, spores, plant debris, epithelial cells, algae, protozoa, viruses and bacteria. They are ubiquitous in the atmosphere [67,68] and cover a very large size range from viruses (about 1 nm diameter) to pollen (up to 300 µm diameter) [69]. A recent campaign over the Caribbean Sea revealed that viable bacterial cells represented on average 20% of the total particles in the 0.25–1 µm diameter range and were at least one order of magnitude more abundant than fungal cells, suggesting that bacteria represented an important and underestimated fraction of micrometre-sized atmospheric aerosols [70]. Bacteria could directly influence the atmospheric chemistry of TEs, for example, through the degradation TE-complexing carboxylic compounds [71,72] and the release of metabolic compounds, such as siderophores [73]. Despite these advances, airborne microorganisms above the oceans remain essentially uncharacterized, as most work to date is restricted to samples taken close to the continents.

Other atmospheric compounds that could complex Fe are humic-like substances (HULIS) [74,75] and/or sugars. These have been detected in rainwater samples and in the water-soluble fraction of aerosol particles (e.g. [55]) and have been shown to bind Fe, at least in the ocean [76].

The expanded range of TEs studied under the GEOTRACES programme not only provides information about additional micronutrients (Zn, Co, Cd, Cu, Ni, etc.), but has also allowed further progress in understanding the solubility behaviour of Fe through the synergies with elements with similar sources or chemistry. For instance, over the spatial scale of the North and South Atlantic Ocean the variation in fractional solubility with total element aerosol concentration of Fe, Al and silicon (Si) has been found to be very similar [77], suggesting that redox processes and anthropogenic inputs are relatively minor controls of aerosol Fe solubility over that spatial scale.

### (c) Biogeochemical impact of trace element deposition

**Figure 3.**
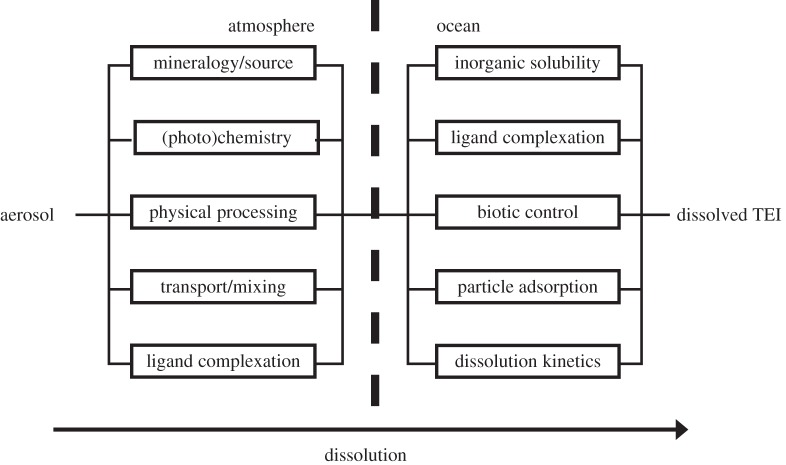
Conceptual model of aerosol TEI solubility controls proposed (for Fe) by Baker & Croot [8], with the addition of a new control factor in the atmosphere: ligand complexation, which may be linked to bioaerosols (see text for more details).

Ultimately, the biogeochemical impact of TEs that enter the ocean via the air–sea interface is dependent on the characteristics of the marine waters into which they are deposited, in addition to the characteristics of the TEs at the point of deposition [78]. The combination of atmospheric and marine influences on solubility has been discussed for Fe by Baker & Croot [8], who suggested a conceptual model of aerosol iron solubility controls in which the various competing and inter-related processes that influence (Fe) solubility in the atmosphere and seawater are likened to electrical resistors connected in parallel in each compartment. In figure 3, we revisit that conceptual model, adding (Fe-) binding ligands in the atmosphere and revising it to describe TE dissolution in general. Whether these newly considered organic ligands result from atmospheric biological activity or have a significant impact on TE solubility in seawater still needs further investigation.

The combined effects of atmospheric and seawater influences on TE dissolution have been studied by addition of mineral dust to natural seawater at scales ranging from bottle incubations to mini- and meso-cosm experiments. For example, an initial addition of dust during the DUNE mesocosm experiment led to decreased dissolved Fe concentrations due to adsorption [79,80]. A second addition of dust to the DUNE mesocosms produced a very different response, with increased dissolved Fe concentrations facilitated by higher Fe-binding ligand concentrations [80]. The percentage of dust Fe released into seawater can be dependent on season and related to surface water-dissolved organic matter concentrations and character [81]. The DUNE experiments, involving the addition of controlled amounts of well-characterized dust to large volumes of isolated *in situ* seawater, have provided the opportunity to study the fate and impact of deposited dust in a manner not possible through either laboratory or field experiments.

The biological response of oceanic waters to dust or ambient aerosol addition has been studied in a number of short-term bottle incubation experiments [82–85]. In many of these experiments, responses to dust addition were different from the systems' responses to the addition of macronutrients (nitrogen (N) and phosphorus (P)) and Fe, or additions of combinations of these. It is apparent that a multi-element approach to such studies is necessary in order to interpret their results [85].

### (d) Non-dust sources of trace elements and their isotopes

Although mineral dust probably constitutes the major atmospheric source of TEIs to the ocean on a global scale, other sources, including volcanic ash [86,87], ship exhaust [88] and land-based anthropogenic emissions [7,89,90], can also be significant on smaller scales. In a similar manner to the behaviour of mineral dust, the deposition of volcanic ash has the potential to decrease surface water Fe concentrations through scavenging, as well as acting as a source of dissolved Fe [91]. Anthropogenic sources of Fe have been found to be significantly more soluble than mineral dust Fe (e.g. [92,93]). Gas-phase emissions from ship exhaust include CO_2_, NO_*x*_ and SO_2_ [88], with the latter two being precursors of atmospheric acidity. Particulate emissions from shipping have been found to contain a number of TEIs including Ni, vanadium (V), lead (Pb), Fe and Zn (e.g. [94–96]). In general, little is known about whether such emissions might have a significant effect on the aeolian delivery of TEIs to the ocean around major shipping routes. However, global ship traffic is projected to increase over the coming decades and one modelling study has indicated that ship emissions might constitute a significant source of soluble Fe to some ocean regions by 2100 [97].

Isotopic data on aerosol TEs may also be useful in distinguishing sources, such as between biomass burning or mineral dust for Fe [11], between anthropogenic emissions or mineral dust for Zn [98] or between combustion aerosols from different regions with Pb isotopes (e.g. [99]).

### (e) Value of coordinated international research programmes

The large amount of new observational data acquired through work by the GEOTRACES and other international research programmes are useful for validation of numerical models and serve to enhance our understanding of TE air–sea interactions. These studies have highlighted the importance of atmospheric transport regimes and deposition modes in determining the overall air–sea flux of TEIs and their impacts on marine biogeochemistry. GEOTRACES data are particularly valuable in this context because the programme's sampling strategy aims to produce a co-collected, corresponding set of TEI data for surface waters.

The collection of a coherent set of TEI data for aerosols through the GEOTRACES programme has been underpinned by the successful aerosol intercalibration/intercomparison exercise [100]. GEOTRACES standardization and intercalibration protocols for oceanic samples generally entail sharing of replicate samples among various laboratories/analysts and sampling at common locations (crossover stations). For atmospheric aerosols, air mass origin and aerosol composition are highly variable, so the applicability of crossover stations is problematic and the best options for aerosol intercalibration are a readily available reference material and/or plentiful marine aerosol sample replicates. During the 2008 GEOTRACES aerosol intercalibration [100], a set of replicate aerosol samples consisting of a mixture of marine, lithogenic and anthropogenic components was successfully analysed for many total element and soluble ion concentrations. It was recommended that digestions for ‘total’ TEI concentrations should use nitric acid, hydrofluoric acid, heat and pressure to achieve total dissolution of aerosol material. The exercise also revealed discrepancies in the measurement of soluble aerosol TEI concentrations, most importantly Fe, a key parameter in many observational and modelling studies.

## 3. Recommendations for further research

### (a) Analytical issues

The continuation and expansion of intercalibration/intercomparison exercises will be necessary to provide coherent datasets for future work. Because aerosols may be analysed for a broad spectrum of TEIs and soluble species (e.g. chloride, nitrate, sulfate and soluble organic compounds), a substantial amount of aerosol material for intercalibration is required. A suitable ‘reference’ material is required to facilitate this intercalibration work. It should be very fine-grained (to mimic aerosol particle sizes), homogeneous at small scales (less than 20 mg) and be readily available at low cost.

— To address the lack of a suitable aerosol certified reference material (CRM), the Arizona Test Dust (ATD) produced by Powder Technology, Inc. is currently being evaluated. ATD is a dry aerosol powder that has been oven dried and sieved, but has not been subjected to washing or leaching. ATD is available in several different size ranges, including A1 Ultrafine (PN 12103-1) whose particle size distribution shows approximately 70% less than 5.5 µm and approximately 98% less than 11 µm. It has a composition very similar to mineral (desert) dust: http://www.powdertechnologyinc.com/product/iso-12103-1-a1-ultrafine-test-dust/. A large quantity has been purchased and our preliminary tests show that it is homogeneous at subsample masses of 10–20 mg.
— A second round of intercalibration tests has begun in 2016; subsamples of the A1 Ultrafine ATD have already been sent to a number of international laboratories to measure total TEI and soluble TEI concentrations, and we hope to recruit additional collaborators for this effort. As part of this intercalibration effort, we also want to encourage the use of ATD for intercomparison of various aerosol solubilization schemes. Our goal is to avoid the cost and time delays needed to produce a true CRM or standard reference material, and to use the ATD material to intercalibrate analysis of aerosol TEIs in much the same way that the SAFe and GT seawater samples have been used to intercalibrate the sampling and analysis for TEIs in seawater. Subsamples of our large batch of the A1 Ultrafine ATD are freely available (contact W.M.L. at wlanding@fsu.edu or P.L.M. at pmorton@fsu.edu).
— Finally, we are also investigating the availability of replicate aerosol samples collected during research cruises to further advance aerosol intercalibration. Members of the international aerosol community are encouraged to facilitate this intercalibration by communicating and collaborating; discussion is underway regarding establishment of a SCOR Working Group on aerosol chemistry and solubility.

### (b) Deposition

— A multi-tracer approach shows promise in reducing the uncertainties associated with quantifying dust deposition fluxes to the ocean [23]. GEOTRACES products are likely to expand the range of tracers and isotopes that can be used for this purpose which should lead to further reduction of this key uncertainty.
— Modelling of dust and TEI deposition to the oceans is an essential part of the study of the Earth system, since it allows estimation of TEI fluxes over spatial and temporal scales which will never be accessible through direct observation. Modelling activities of this nature are inherently uncertain, however, because they inevitably involve the simplification of highly complex systems that are themselves incompletely understood. The modelling community conducts occasional intercomparison exercises [101,102] in order to assess the variability between models and to aid in model development. Since the ultimate product of these models is the deposition flux of TEIs to the ocean, it makes sense for the intercomparison exercises to report comparisons of deposition flux. We note, however, that comparison to observations is also an important part of validation and development of models. We would therefore suggest that future model intercomparisons should also report model mean aerosol surface-level concentrations, since these are directly available from observations, whereas deposition fluxes to the ocean are not.

### (c) Solubility

Ultimately, we wish to provide the modelling community with sufficient information to include realistic descriptions of TE solubilization in numerical models in the most computationally efficient manner possible. In order to do this, we need to clarify several outstanding issues.

— To what extent do anthropogenic emissions influence the solubility of TEI in aerosols? (How significant, on global and regional scales, are direct anthropogenic emissions of TEIs in determining the overall deposition of soluble forms of those TEIs to the ocean? What is the indirect impact of anthropogenic emissions of acidic (NO_*x*_, SO_2_) and alkaline (NH_3_) substances on the evolution of aerosol TEI solubility during atmospheric transport?)
— Further improvement in our understanding of the influence of organic matter and bioaerosols on TEI solubility in aerosols and in seawater after deposition, as well as their potential impact on bioavailability, is required.
— Deposition of aerosol particles and rainfall to the surface ocean requires the transfer of material across the sea surface microlayer (SSM), a region whose biogeochemical characteristics are quite distinct from the properties of bulk seawater [103]. In all probability, the SSM has a significant influence on TEI solubility, but our understanding of the extent of this influence is still in its infancy.
— Similarly, conditions in bulk seawater will also have a significant (perhaps dominant for Fe) influence on TE solubility. We still need to improve our understanding of TEI dissolution ‘length scales’ and kinetics in relation to particle residence times in the ocean. Some of that understanding may only be accessible via process studies or meso-scale oceanic enrichment experiments.

### (d) Anthropogenic impacts

— There has been much recent interest in the role played by anthropogenic emissions in introducing TEIs into the atmosphere (e.g. for soluble Fe, as stated above). Characterization of exemplar source end-members for these emissions will be required in order for them to be incorporated into numerical models. Emissions from shipping are of particular interest in this context as shipping has seen rapid growth in recent decades and this growth is projected to increase further into the near future. Elements such as Ni and V are of particular concern with regard to ship emissions, as are acid precursors (NO_*x*_ and SO_2_), although changes in regulations relating to ship emissions may influence this [104,105].
— The introduction of routine sampling for black carbon (QMA filters) into GEOTRACES protocols will help to link aerosol TEI concentrations to anthropogenic emissions and will also aid in the validation of anthropogenic emissions and transport in numerical models.

Although our paper has focused primarily on studies conducted under the international GEOTRACES programme, we note that other international scientific programmes (e.g. SOLAS) share many of the goals of GEOTRACES. We encourage the development of links between these programmes through the sharing of data and expertise. For example, the SOLAS Aerosol and Rainwater Chemistry database (http://www.bodc.ac.uk/solas_integration/implementation_products/group1/aerosol_rain/) contains a large amount of GEOTRACES-relevant data and open access to results obtained by both programmes is of clear benefit to both communities.
